# Development and validation of LC/MS/MS method for the simultaneous determination of montelukast, gliclazide, and nifedipine and its application to a pharmacokinetic study

**DOI:** 10.1186/1752-153X-8-17

**Published:** 2014-03-11

**Authors:** Essam Ezzeldin, Nisreen F Abo-Talib, Marwa H Tammam, Abdelaaty A Shahat

**Affiliations:** 1 Drug Bioavailability Lab.College of Pharmacy, King Saud University, P.O. Box 2457, Riyadh 11451, Saudi Arabia; 2 Drug Bioavailability Center, National Organization for Drug Control and Research, P.O.Box 29, Cairo, Egypt; 3 Department of Pharmacognosy, College of Pharmacy, King Saud University, P.O. Box 2457, Riyadh 11451, Saudi Arabia

## Abstract

**Background:**

Montelukast is a leukotriene receptor antagonist for treatment of asthma, gliclazide is an oral hypoglycemic antidiabetic agent, and nifedipine is a calcium channel blocker used for treatment of angina pectoris and hypertension. These drugs may be prescribed to patients suffering from these chronic diseases. A survey of the literature reveals that there is no reported method for the simultaneous determination of montelukast, gliclazide, and nifedipine in pharmaceutical preparations or biological fluids.

**Results:**

A simple, sensitive, and rapid method for the simultaneous quantification of montelukast, gliclazide, and nifedipine in human plasma was developed and validated. Montelukast, gliclazide, and nifedipine were resolved using rapid resolution LC/MS/MS Agilent system and SB-C_18_ (50 × 4.6 mm) 1.8 μm particle size column. The mobile phase consisted of acetonitrile: 0.1% formic acid (84:16). The three drugs were simultaneously extracted from plasma by protein precipitation with acetonitrile using zaferolukast as an internal standard. The method was validated according to FDA guidelines with good reproducibility and linearity of 0.999 and the limits of quantification were 0.11, 0.04, and 0.07 ng/mL for montelukast, gliclazide, and nifedipine, respectively. The accuracies of the three QCs for the three drugs were 99.48% (montelukast), 106.53% (gliclazide), and 108.03% (nifedipine) in human plasma. The validated method was applied to a pharmacokinetic study in human volunteers after oral administration of the three drugs. The applied LC/MS/MS method was shown to be sufficiently sensitive and suitable for pharmacokinetic studies.

**Conclusion:**

The LC/MS/MS method was validated and successfully applied for the determination of montelukast, gliclazide, and nifedipine concentrations in human plasma.

## Background

Montelukast, gliclazide, and nifedipine are drugs used for the management of chronic asthma and as prophylactic agents for exercise-induced asthma, treatment of non-insulin-dependent diabetes mellitus, and treatment of angina pectoris and hypertension, respectively.

This study aimed to determine serum concentrations of these three drugs, which are often jointly prescribed to patients with chronic diseases such as asthma, hyperglycemia, and hypertension.

**Figure 1:**
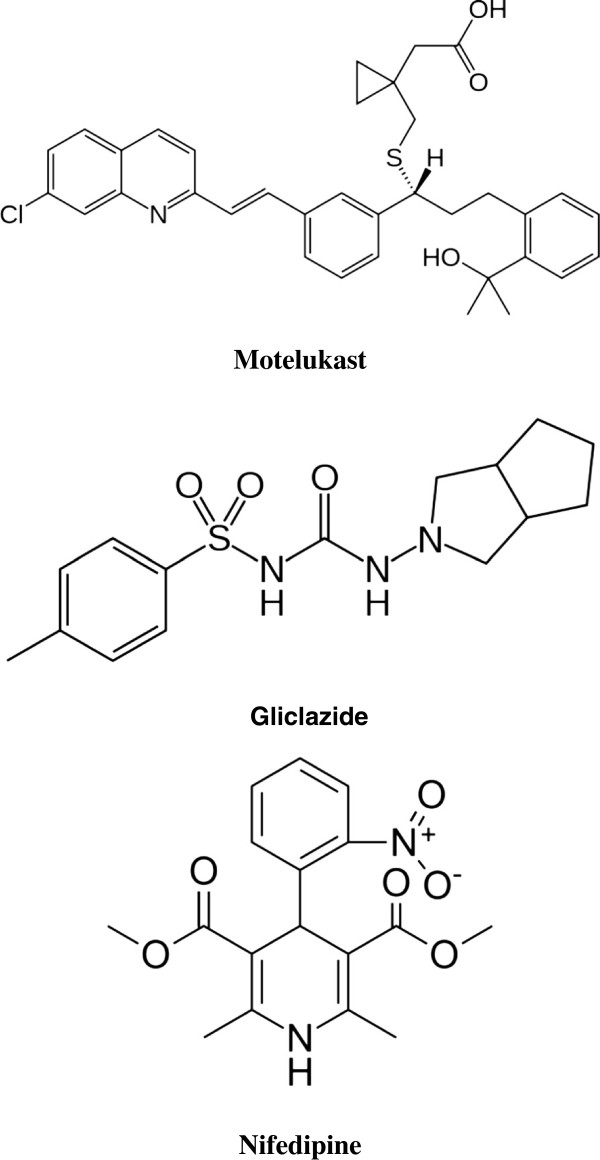
Chemical structures of montelukast, gliclazid and nifedipine.

Montelukast sodium (MO), 1-[({(R)-m-[(E)-2-(7-chloro-2-quinolyl)-vinyl]-α-[o-(1-hydroxy-1-methylethyl) phenethyl]-benzyl}thio)methyl]cyclopropane acetate (Figure 1) is a selective leukotriene receptor antagonist for uses similar to those of zafirlukast, although it is reported to have a longer duration of action [1]. Gliclazide (GL), *N*-(4-methylbenzenesulfonyl)-*N*- (3-azabicyclo- [3.3.0] oct-3-) urea (Figure 1) is a second-generation sulfonylurea [2]. Nifedipine (NI), dimethyl-1,4-dihydro-2,6-dimethyl-4-(2-nitrophenyl)pyridine-3,5-dicarboxylate, is a calcium channel blocker that inhibits the trans-membrane influx of Ca^2+^ into cardiac muscle cells and vascular smooth muscle through specific ion channels [3-5].

Several methods have been developed to quantitatively estimate MO concentration, including voltammetry [6], capillary electrophoresis [7], spectroflurometry [8], spectrophotometry [9], and liquid chromatography (LC) [10-19]. Some of these methods were developed to monitor pharmaceutical dosage forms [14-17] and drug concentrations in biological fluids [8-13,18,19].

On the other hand, GL concentrations have been individually determined using spectrophotometry [20-22], radioimmunoassay [23], gas chromatography [24], HPLC [25-28], and mass spectroscopy [29,30]. In addition, GL concentrations have been determined in mixtures with metformin [31-34], metformin and pioglitazone [35], and rosiglitazone [36].

A detailed survey of the literature for NI revealed several reported methods for the assay of NI either alone or in combined drug formulations. These analytical techniques include spectrophotometry [37-42], HPLC [43-47], high performance thin layer chromatography [48], micellar electrokinetic chromatography [49], electroanalytical methods [50,51], flow injection analysis [52], and mass spectrometry [53,54].

The present study aimed to develop a simple, sensitive, reproducible, and rapid LC/MS/MS method to monitor plasma levels of montelukast, gliclazide, and nifedipine for application in pharmacokinetic studies and routine clinical practice.

### Experimental

#### Chemicals and materials

MO standard material (99.5% potency) and zaferolukast (ZA) (I.S.) (99.3% potency) were kindly supplied by Merck. GL and NI standard material (99.22% and 99.8% potency, respectively) was kindly supplied by Sigma-Aldrich Co. (UK). Singulair 10 mg tablets (MO) (Merk&Co. Inc-USA), Diamicron 80 mg tablets (GL) (Servier), and Epilat retard 20 mg tablets (NI) by EPICO, Egypt.

Formic acid was purchased from Romil chemicals, England) and acetonitrile and methanol (HPLC grade) were purchased from, Alpha Chemicals, Egypt). Deionized water was obtained from a Milli-Q water purification system 3 (Millipore, France), and human plasma was supplied by VACSERA, Egypt). The mobile phase was filtered through a 0.45-μm Whatman membrane filter.

### Instruments and chromatographic conditions

An Agilent triple quadrupole mass spectrometer with an API source coupled with an Agilent pump controlled by an Agilent 1200 controller and equipped with an Agilent 1200 autosampler injector was used for analysis. Separation was performed using an analytical Agilent SB-C18 column (50 × 4.6 mm) with particle size of 1.8 μm. The mobile phase consisting of acetonitrile: 0.1% formic acid (84:16), was delivered at a flow rate of 0.6 mL/min.

Mass spectra were obtained using an electrospray ionization source operated in the multiple reaction monitoring (MRM) mode. Sample introduction and ionization were both performed in the positive ion mode (MO, GL, and NI) and negative ion mode (ZA). The cone voltage was set at 135 V for both MO and GL and 80 V and 150 V for NI and ZA, respectively. The capillary voltage was optimized at 4000 V. Argon was used as the collision gas. The collision energy was set at 20, 15, 5, and 25 MeV for MO, GL, NI, and ZA, respectively. Optimal gas flow during tuning was 8 L/min and nebulizer pressure was 30 psi. The source temperature was 325°C. The selected mass transitions ion pairs were 586.2/568.3, 324.4/127.2, 347.3/315.2 at (positive ion) and 574.6/462.2 at (negative ion) for MO, GL, NI, and ZA (I.S.), respectively. Agilent Mass Hunter software was used for data acquisition. For quantification, the peak area ratios of the target ions of the drugs to those of the internal standard were compared with weighted (1/concentration^2^) least square calibration curves in which the peak area ratios of the calibration standards were plotted versus their concentrations.

### Preparation of stock solutions and calibration standards

Stock solutions of each of the MO, GL, NI, and ZA (I.S.) samples were prepared in methanol at a concentration of 100.0 μg/mL and stored at 4°C. Handling and analysis of all samples were performed under diffused light conditions (prepared away from light using opaque glasses and aluminum foil). Evaluations of the assay were performed by seven point calibration curves made by serial dilutions of the stock solution of each drug at the nominal concentration ranges of 10.0–800.0 ng/mL, 10.0–5000.0 ng/mL, and 10.0–600.0 ng/mL for MO, GL, and NI, respectively, in human plasma. The slopes and intercepts of the calibration lines were determined.

### Sample preparation

Sample preparation was performed by liquid–liquid extraction of 100 μL of plasma by protein precipitation using 300 μL of acetonitrile. A 5.0-μL aliquot of the internal standard solution was added. The mixture was then shaked by vortex for 30 s and centrifuged for 10 min at 4000 rpm. A 20 μL aliquot of the supernatant was injected into the LC/MS/MS instrument.

### Clinical protocol

This method was applied for the analysis of plasma samples after the administration of a single dose of 10 mg MO (Singulair tablets), 80 mg GL (Diamicron tablets), and 20 mg NI (Epilat retard tablets) to six healthy male volunteers. The study protocol was approved by the Ethics Committee of Bioavailability Studies (NODCAR). The age of the volunteers ranged from 29 to 39 years and the body weights of the subjects ranged from 58 to 71 kg. All subjects provided written informed consent. The study was conducted in accordance with the provisions of the Declaration of Helsinki. After an overnight fast for 10 h, all volunteers received a single dose of the three drugs with 200 mL of water. Blood samples (3 mL) from a suitable antecubital vein were collected into heparin-coated tubes at 0.0 (before dose), 0.25, 0.5, 0.75, 1.0, 1.5, 2.0, 2.5, 3, 4, 6.0, 8.0, 10.0, 12.0, 24.0, and 48.0 h post dosing. The blood samples were centrifuged at 3000 rpm for 5 min at room temperature and the plasma was removed and stored at −80°C until assayed for MO, GL, and NI content. All samples from a single volunteer were analyzed in the same run in order to avoid inter-assay variations.

### Pharmacokinetic analysis

Pharmacokinetic parameters from the human plasma samples were calculated by a noncompartmental model using the WinNonlin 5.3 software. Blood samples were taken for a period of 48 h. and it was considered as the area under the concentration–time curve (AUC) ratios were higher than 80% as per FDA guidelines [55,56]. The first-order terminal elimination rate constant (K_el_) was estimated by linear regression from the points describing the elimination phase on a log-linear plot. The maximum observed plasma concentration (C_max_) and the time taken to achieve this maximum level (t_max_) were directly obtained from the curves.

The areas under the time-concentration curve for MO, GL, and NI plasma concentration versus time for 48 h (AUC_0–48_) were calculated using the trapezoidal method. Extrapolation of this area to infinity (AUC_0–∞_) was performed by adding the value C_48_/K_el_ to the calculated AUC_0–48_ where C_48_ is the MO, GL, and NI plasma concentration at 48 h. K_el_ is the first-order terminal elimination rate constant.

## Results and discussion

New methods for simultaneous determination of two or more compounds without interference from each other are always of interest. The goal of this work was to develop and validate a simple, rapid, and sensitive LC/MS/MS assay method for simultaneous extraction and quantification of montelukast, gliclazide, and nifedipine in plasma and to apply this method in a pharmacokinetic study. To achieve these goals, during method development, different options were evaluated to optimize sample extraction, detection parameters, and chromatography.

### Method development

LC conditions were optimized to obtain a short run time and adequate resolution between MO, GL, NI, and the internal standard ZA. Several trials were performed to select an optimal ratio of acetonitrile to 0.1% formic acid. These trials determined that 86% acetonitrile ratio was optimal for simultaneous separation of the three drugs. Using this mobile phase delivered at a flow rate of 0.6 mL/min resulted in improved signals when compared with different ratios of same reagents. In addition, MS parameters were optimized to obtain maximum sensitivity.

**Figure 2:**
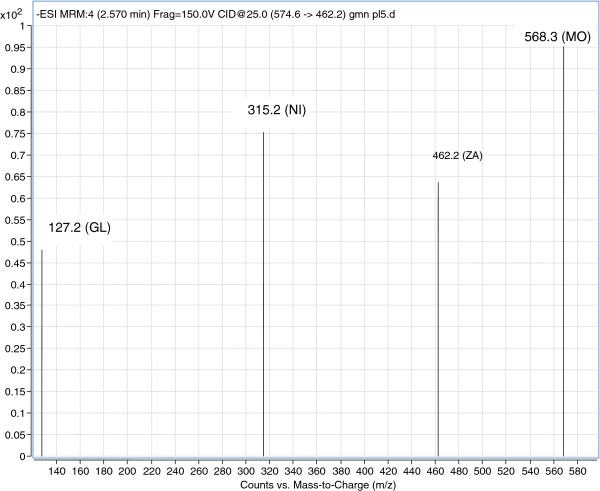
Mass spectra of the positive ion of montelukast (MO), gliclazide (GL), nifedipine (NI) and negative ion zaferolukast (ZA) internal standard.

**Figure 3:**
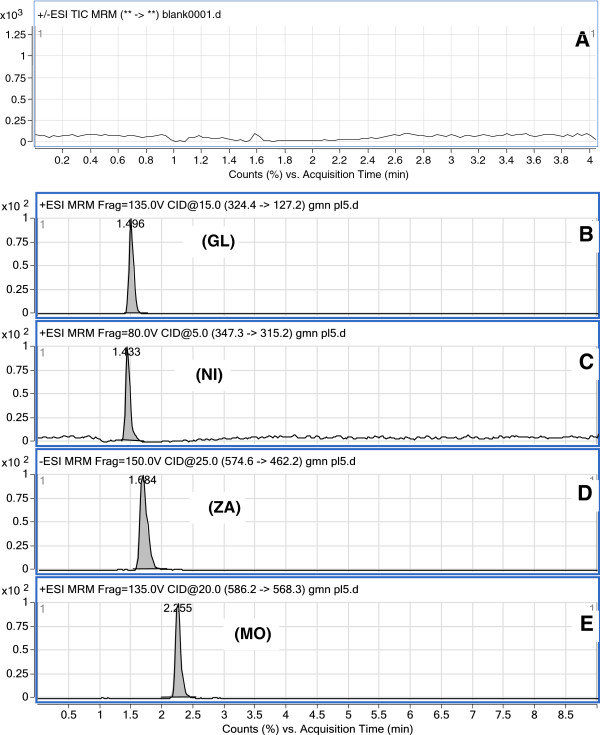
MRM chromatograms of blank plasma (A), gliclazide (B), nifedipine (C), zaferolukast (D) and montelukast (E).

Electrospray ionization (ESI) was evaluated to obtain a better response from the analytes. The best signal was achieved using an ESI-positive ion mode for MO, GL, and NI, and negative ion mode for ZA. The product ion spectrum for MO, GL, NI, and ZA yielded a high abundance of fragment ions of m/z 568.3, 127.2, 315.2, and 462.2, respectively (Figure 2). The representative extracted ion chromatograms of the three drugs and the internal standard are shown in Figure 3.

### Method validation

#### Selectivity, linearity, limit of detection, and limit of quantitation

The selectivity of the method was evaluated by comparing the chromatograms obtained from the MO, GL, and NI samples and the internal standard with those obtained from the blank samples. A representative chromatogram obtained from blank plasma is shown in Figure 3A. The chromatograms of the spiked plasma samples are shown in Figure 3B, C, D, and E. Under the optimized conditions, a linear relationship with a good correlation coefficient (r = 0.999, n = 6) was observed between the peak area ratios and the concentrations of MO, GL, and NI in the range of 10.0–800.0, 10.0–5000.0, and 10.0–600.0 ng/mL, respectively. The experiments were performed using a standard seven-point series for each drug.

The limit of detection (LOD) and limit of quantitation (LOQ) were calculated according to the ICH guidelines for validation of analytical procedures based on the standard deviation of the response and the slope of the calibration curve [57] using the formula: LOD or LOQ = κσ/S, where κ = 3.3 for LOD and 10 for LOQ, σ is the standard deviation of the response, and S is the slope of the calibration curve. Calculations of six replicate experimental injections determined LODs of 0.04, 0.01, and 0.02 ng/mL and LOQs of 0.11, 0.04, and 0.07 ng/mL for MO, GL, and NI, respectively.

#### Accuracy and precision

The accuracy and precision of the proposed method were determined by intra-day and inter-day replicate analyses of plasma spiked with different concentrations of each drug covering the working linear ranges. The inter-day assays were carried out on three different days with the same concentration levels for spiked plasma samples. The accuracy values from the intra-day analysis were 94.59% –105.58%, 89.75% −114.86%, and 83.46%–110.22%, whereas the values for the inter-day analysis were 93.11%, −109.32%, 99.93%–106.16%, and 97.90%–114.82% for MO (Table 1), GL (Table 2), and NI (Table 3), respectively, indicating the accuracy and precision of the proposed method. The volunteer samples that had greater concentrations compared with the highest concentration in the validation were diluted before injection and the actual concentrations were recalculated.

**Table 1:** Intra-day and inter-days precision and accuracy for determination of montelukast in spiked human plasma

	**Theoretical concentration (ng/mL)**													
**Parameters**	**Intra-day reproducibility**							**Inter-days reproducibility**						
	**10**	**20**	**40**	**80**	**120**	**400**	**800**	**10**	**20**	**40**	**80**	**120**	**400**	**800**
**Concentration found (ng/mL)**														
**Mean**	10.93	19.9	38.5	77.43	109.16	372.45	808.55	10.37	18.92	37.95	78.23	105.58	392.03	816.69
**Precision**	1.69	7.79	4.49	8.73	4.56	14.13	7.68	13.74	12.52	14.01	13.81	11.34	14.14	12.84
**Accuracy (%)**	109.32	99.5	96.24	96.79	109.16	93.11	101.07	103.66	94.59	94.89	97.79	105.58	98.01	102.09

**Table 2:** Intra-day and inter-days precision and accuracy for determination of gliclazide in spiked human plasma

	**Theoretical concentration (ng/mL)**													
**Parameters**	**Intra-day reproducibility**							**Inter-days reproducibility**						
	**10**	**20**	**100**	**250**	**500**	**1000**	**5000**	**10**	**20**	**100**	**250**	**500**	**1000**	**5000**
**Concentration found (ng/mL)**														
**Mean**	11.49	18.5	89.75	241.79	512.61	1045.04	4767.74	10.27	19.99	106.16	261.21	527.12	1037.07	5215.71
**Precision**	7.35	11.1	13.49	11.42	5.33	0.6	10.92	15.54	15.17	15.39	14.92	4.17	3.22	10.49
**Accuracy (%)**	114.86	92.5	89.75	96.72	102.52	104.5	95.35	102.68	99.93	106.16	104.48	105.42	103.71	104.31

**Table 3:** Intra-day and inter-days precision and accuracy for determination of nifedipine in spiked human plasma

	**Theoretical concentration (ng/mL)**													
**Parameters**	**Intra-day reproducibility**							**Inter-days reproducibility**						
	**10**	**20**	**40**	**80**	**100**	**200**	**600**	**10**	**20**	**40**	**80**	**100**	**200**	**600**
**Concentration found (ng/mL)**														
**Mean**	8.35	21.33	44.09	83.93	101.1	203.63	634.48	10.51	20.01	39.16	91.17	114.82	227.41	664.38
**Precision**	4.21	10.55	6.69	8.09	4.2	2.76	1.45	8.84	5.7	15.54	14.52	4.86	1.82	2.47
**Accuracy (%)**	83.46	106.67	110.22	104.91	101.1	101.82	105.75	105.08	100.04	97.9	113.96	114.82	113.7	110.73

#### Application to pharmacokinetic study

**Figure 4:**
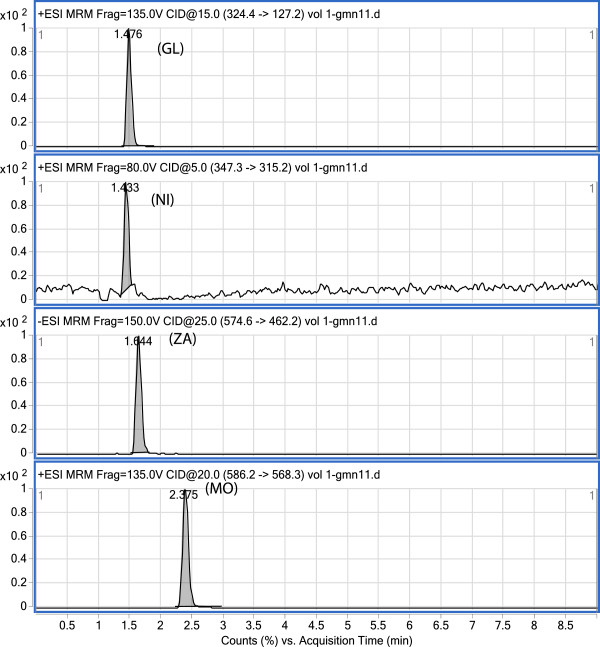
MRM chromatogram of Gliclazide (GL), Nifedipine (NI), zaferolukast (ZA), and Motelukast (MO) extracted from plasma sample of healthy volunteer (Subject No. 2, at 6 h.).

**Figure 5:**
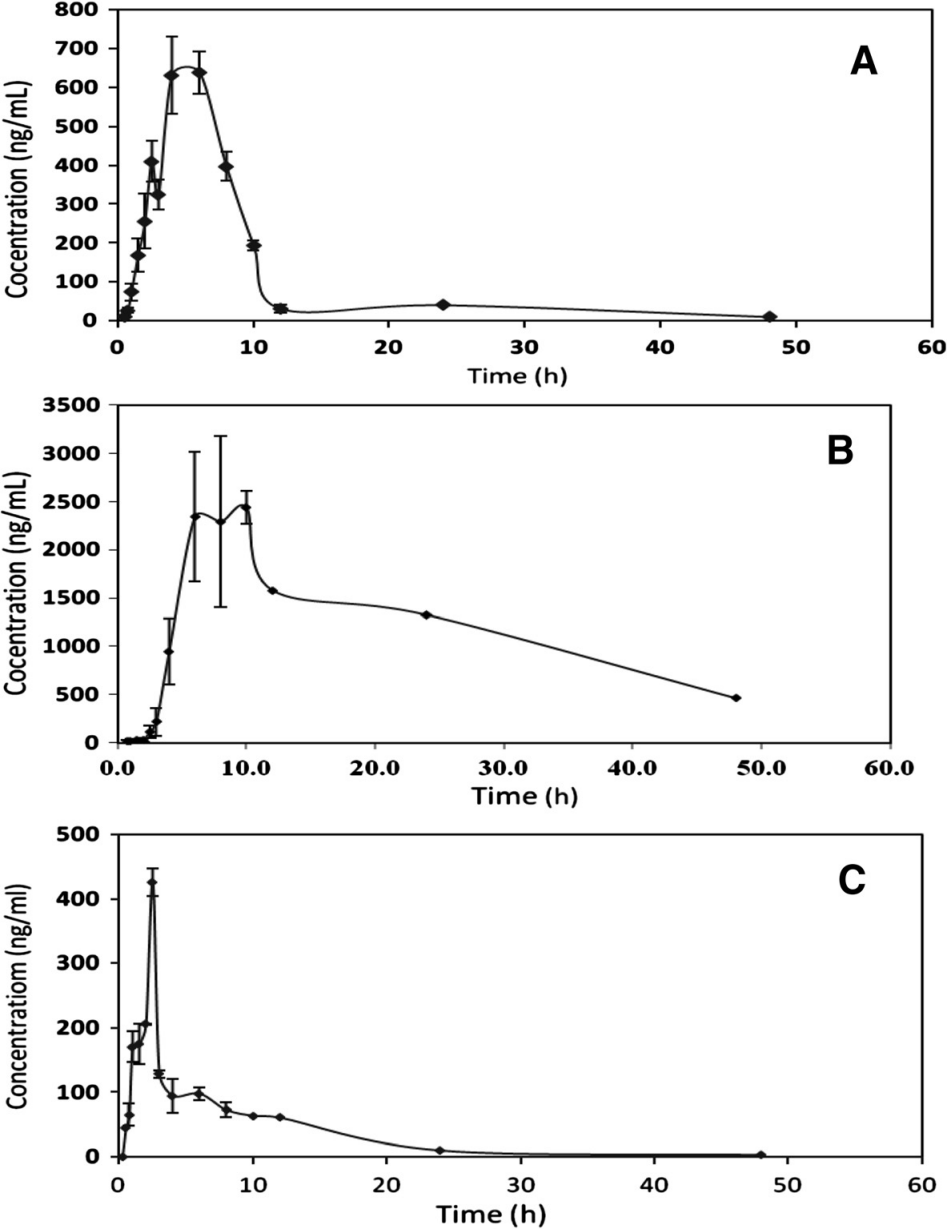
Mean (±SD) Plasma Concentration versus time of montelukast (A), gliclazide (B) and nifedipine (C) following simultaneous administration of montelukast (10 mg), gliclazide (80 mg) and nifedipine (20 mg) Tablets to six healthy male volunteers.

The method described here was successfully applied to a pharmacokinetic study of MO, GL, and NI in plasma samples from healthy human volunteers. None of the volunteers exhibited any general adverse reactions. Following oral drug administration, plasma concentration–time profiles of the three drugs best fit a non-compartment in all subjects. A representative MRM chromatogram of a plasma sample extracted from a healthy volunteer at 6 h is shown in Figure 4. A representative plasma concentration–time profile is shown in Figure 5. The estimated pharmacokinetic parameters are shown in Table 4.

**Table 4:** Pharmacokinetic Parameters required for assessment of Montelukast, Gliclazide and Nifedipine Bioavailability

**Parameters**	**Montelukast**	**Gliclazide**	**Nifedipine**
C_max_ (ng/mL)	882.39 ± 245.8	2917.2 ± 991.3	353.519 ± 104.5
AUC _0–48_ (ng.h/mL)	4493.23 ± 2576.3	45156.15 ± 45988.13	1261.81 ± 155.1
AUC _0-α_ (ng.h/ml)	4797.49 ± 2808.6	49915.8 ± 51881.3	1423.3 ± 295.5
t_max_ (median) h	4.25	8.00	4.24
t_1/2_ (h)	3.86 ± 27	7.24 ± 8.3	2.7 ± 0.36
MRT (h)	7.27 ± 3.7	15.46 ± 11.6	5.88 ± 3.1
K_el_ (h)	0.282 ± 0.32	0.240 ± 0.17	0.255 ± 0.03

## Conclusions

The results of this study showed that the validated LC/MS/MS method proved to be a simple, rapid, reliable, selective, and sensitive method sufficient for simultaneous monitoring of pharmacokinetic parameters of montelukast, gliclazide, and nifedipine. A small plasma sample volume and LOQ were sufficiently sensitive to detect terminal phase concentrations of the drugs.

## Abbreviations

MO: Montelukast sodium; GL: Gliclazide; NI: Nifedipine; LC/MS/MS: Liquid chromatography/Mass/Mass spectrometry; LOD: Limit of detection; LOQ: Limit of quantification; I.S: Internal standard.

## Competing interests

The authors declare that they have no competing interests.

## Authors’ contributions

EE proposed the subject, designed the study, conducted pharmacokinetic and statistical analysis, participated in the results discussion and revised manuscript. NFA participated in study design, assay design, literature review, conducted the validation of the assay, analysis of the samples, participated in the results discussion and participated in preparing the manuscript. MHT participated in study design, assay design, literature review, conducted the validation of the assay, analysis of the samples, participated in the results discussion and participated in preparing the manuscript. AAA participated in the writing the manuscript. All authors read and approved the final manuscript
